# CRISPR Contributes to Adhesion, Invasion, and Biofilm Formation in Streptococcus agalactiae by Repressing Capsular Polysaccharide Production

**DOI:** 10.1128/spectrum.02113-21

**Published:** 2022-07-21

**Authors:** Meng Nie, Yuhao Dong, Qing Cao, Dan Zhao, Shuting Ji, Hao Huang, Mingguo Jiang, Guangjin Liu, Yongjie Liu

**Affiliations:** a Joint International Research Laboratory of Animal Health and Food Safety, College of Veterinary Medicine, Nanjing Agricultural University, Nanjing, Jiangsu, China; b Guangxi Key Laboratory for Polysaccharide Materials and Modifications, School of Marine Sciences and Biotechnology, Guangxi University for Nationalities, Nanning, China; Institut Pasteur

**Keywords:** *Streptococcus agalactiae*, CRISPR, capsular polysaccharide, adhesion, invasion, biofilm formation

## Abstract

The clustered regularly interspaced palindromic repeat (CRISPR)-associated (Cas) system functions classically as a prokaryotic defense system against invading mobile genetic elements, such as phages, plasmids, and viruses. Our previous study revealed that CRISPR deletion caused increased transcription of capsular polysaccharide (CPS) synthesis-related genes and severely attenuated virulence in the hypervirulent piscine Streptococcus agalactiae strain GD201008-001. Here, we found that CRISPR deficiency resulted in reduced adhesion, invasion, and biofilm formation abilities in this strain by upregulating the production of CPS. However, enhanced CPS production was not responsible for the attenuated phenotype of the ΔCRISPR mutant. RNA degradation assays indicated that inhibited transcription of the *cps* operon by CRISPR RNA (crRNA) was not due to the base pairing of the crRNA with the *cps* mRNA but to the repression of the promoter activity of *cpsA*, which is a putative transcriptional regulator of the capsule locus.

**IMPORTANCE** Beyond protection from invading nucleic acids, CRISPR-Cas systems have been shown to have an important role in regulating bacterial endogenous genes. In this study, we demonstrate that crRNA inhibits the transcription of the *cps* operon by repressing the activity of promoter P*cpsA*, leading to increases in the abilities of adhesion, invasion, and biofilm formation in S. agalactiae. This study highlights the regulatory role of crRNA in bacterial physiology and provides a new explanation for the mechanism of crRNA-mediated endogenous gene regulation in S. agalactiae.

## INTRODUCTION

The clustered regularly interspaced palindromic repeat (CRISPR)-associated (Cas) system is a prokaryotic defense system against foreign genetic elements, such as phages, plasmids, and transposons (1, 2). The CRISPR-Cas locus is typically composed of an operon encoding Cas proteins and a repeat-spacer array consisting of identical repeat sequences and invader-targeting spacer sequences (3). Foreign nucleic acids are recognized by direct hybridization of small CRISPR RNAs (crRNAs), which act in conjunction with Cas proteins to mediate cleavage of the target. CRISPR-Cas components are widely distributed in approximately 50% of bacteria and nearly 90% of archaea (4). Increasing evidence has indicated that in addition to playing a role in adaptive immunity, the CRISPR-Cas system has broader functions in bacterial physiology (5, 6). For example, the type II-B CRISPR-Cas system is required for intracellular infection of *Legionella* in host cells (7), and loss of the CRISPR-Cas system in Neisseria meningitidis (8) or Campylobacter jejuni (9) decreases bacterial ability to attach to, invade, and replicate within host cells. CRISPR-associated protein 9 (Cas9), an endonuclease known for its role in CRISPR-Cas immunity, has also been observed to modulate the colonization and virulence of Streptococcus agalactiae (10).

S. agalactiae, also known as group B *Streptococcus* (GBS), is an opportunistic Gram-positive bacterium that can infect a variety of hosts, including humans, other terrestrial animals, and aquatic species (11). Sialic acid capsular polysaccharide (CPS) is an essential virulence factor of S. agalactiae that can interfere with host opsonophagocytic clearance mechanisms and limit the activation of the innate immune response (12, 13). The CPS of S. agalactiae is composed of oligosaccharide repeating units and terminal sialic acid residues (14). Sialic acid facilitates GBS evasion of immune reactions by interacting with the host immune receptors sialic acid binding immunoglobulin-like lectins (Siglecs) (15, 16). The regulation of the capsule operon in S. agalactiae is poorly studied and understood. The CovR/S system, a major regulator of virulence, has been suggested to activate the expression of *cps* genes (17). Recently, chromatin immunoprecipitation followed by sequencing (ChIP-seq) analysis indicated that CovR could bind to the middle and the 3′ end of the capsule operon (18). Hanson et al. (19) demonstrated that CpsA positively regulates the expression of the capsule by binding to the *cps* operon promoter. However, this result was challenged by a previous study showing that CpsA was not essential for the biosynthesis of the capsular polysaccharide repeating unit but was involved in controlling CPS elongation and attachment to the cell wall (20).

The strain GD2008-001 was isolated from tilapia with meningoencephalitis, which was responsible for the streptococcosis outbreak in Guangdong Province in 2008 (21). Our previous studies demonstrated that this bacterium harbored a single type II-A CRISPR-Cas system (22) and that deletion of CRISPR caused significantly upregulated expression of the CPS synthesis-related genes *cpsB*, *cpsC*, and *cpsJ* (23). In this study, we found that crRNA negatively regulated the production of CPS and thus enhanced the abilities of adhesion to and invasion of brain endothelial cells and biofilm formation. Furthermore, we demonstrated that crRNA did not directly cleave mRNA of the *cps* operon but regulated CPS synthesis by inhibiting the promoter activity of *cpsA*.

## RESULTS

### CRISPR is negatively involved in the production of CPS.

**FIG 1:**
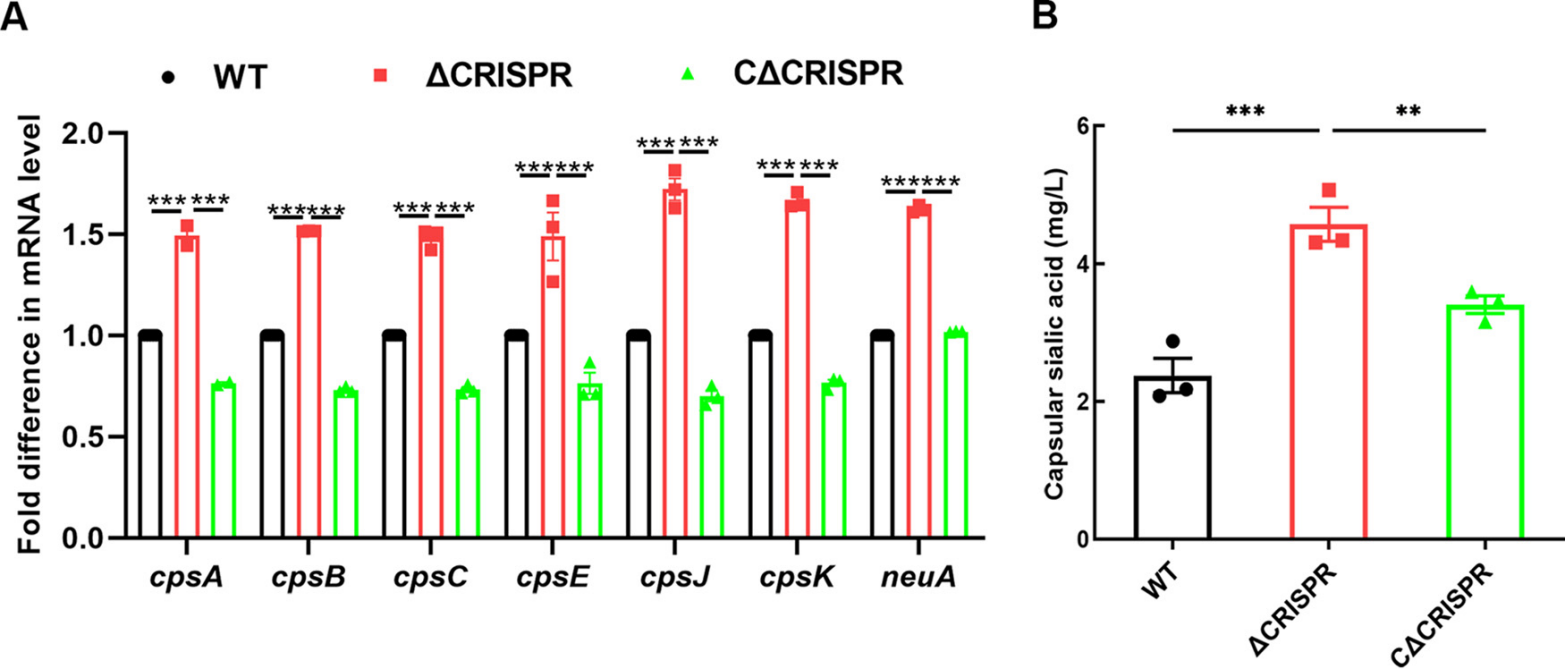
CRISPR negatively affected the production of CPS. (A) Transcription analysis of capsule synthesis-associated genes by qRT-PCR; (B) production of capsular sialic acid in the WT and ΔCRISPR mutant strains. The data are shown as the means ± standard errors of the means (SEM) from three independent experiments. **, *P* < 0.01; ***, *P* < 0.001.

To determine whether crRNA could regulate the transcription of the *cps* operon in S. agalactiae, we detected the expression profiles of the 7 genes randomly selected from the *cps* operon by quantitative reverse transcription-PCR (qRT-PCR). As anticipated, all 7 genes located in the *cps* operon were upregulated in the CRISPR array knockout mutant strain **(**ΔCRISPR**)** compared to its complement strain **(**CΔCRISPR**)** and the wild-type (WT) strain (Fig. 1A), although the upregulation levels of *cpsB*, *cpsC*, and *cpsJ* were 1.52-, 1.48-, and 1.72-fold, respectively, not as notable as those obtained by transcriptome sequencing (RNA-seq), showing increases of 2.50-, 2.88-, and 2.58-fold (see Table S2 in the supplemental material), compared to WT. We then estimated the contents of sialic acid, an important component of CPS, in the WT, ΔCRISPR, and CΔCRISPR strains and found that the ΔCRISPR mutant had significantly higher levels of sialic acid than the WT and CΔCRISPR strains (Fig. 1B). These findings indicated that CRISPR negatively affected the production of CPS by downregulating the *cps* operon.

### Altered surface properties in the ΔCRISPR mutant are closely related to the enhanced production of CPS.

**FIG 2:**
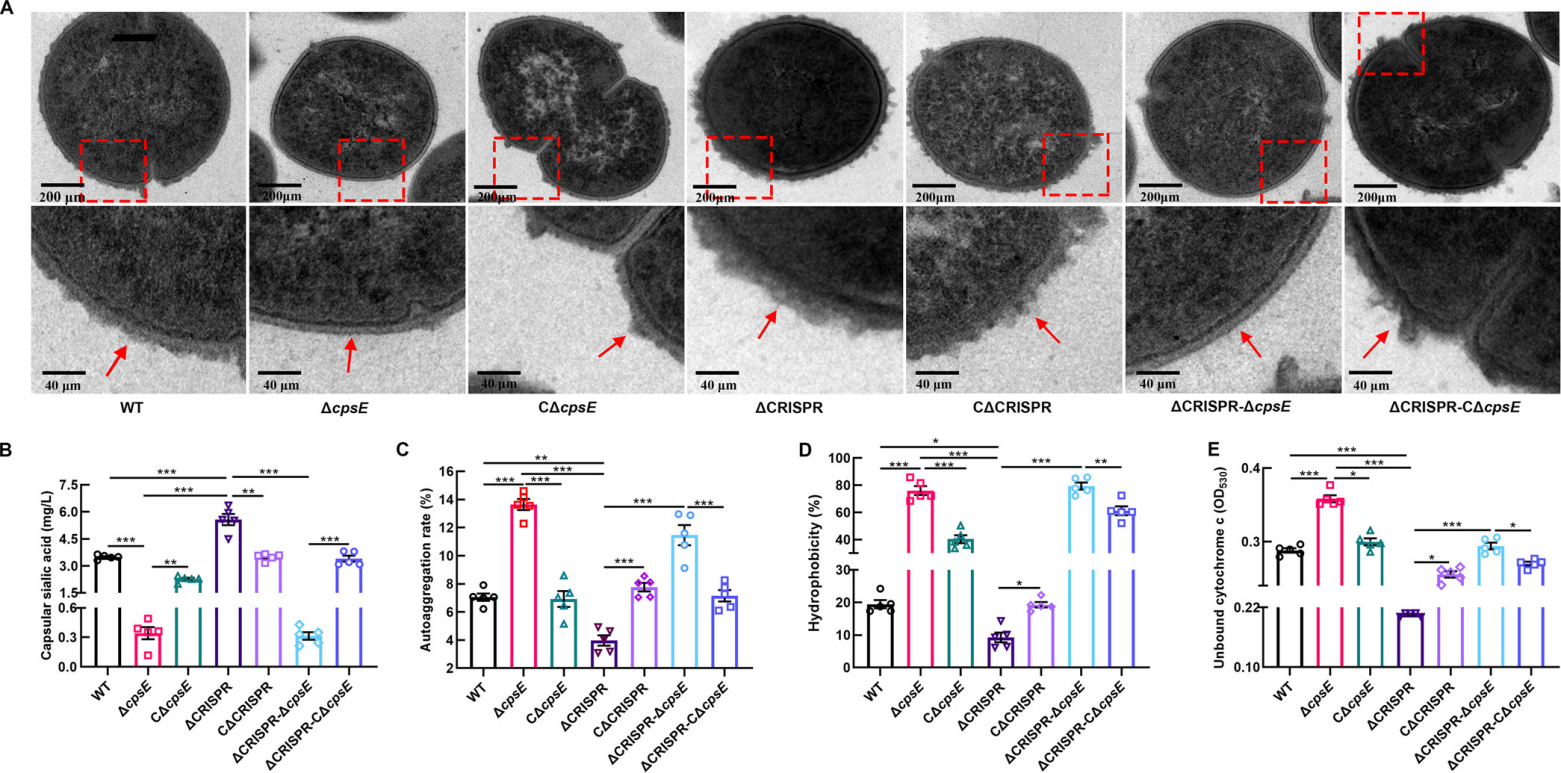
Surface physicochemical properties associated with CPS were regulated by CRISPR. (A) TEM images of the capsule of the WT, Δ*cpsE*, CΔ*cpsE*, ΔCRISPR, CΔCRISPR, ΔCRISPR Δ*cpsE*, and ΔCRISPR CΔ*cpsE* strains. The red arrow indicates the capsule. (B) Production of capsular sialic acid in the indicated strains; (C) autoaggregation of the indicated strains; (D) surface hydrophobicity of the indicated strains; (E) surface anionic charge of the indicated strains. The data are shown as the means ± SEM from three independent experiments. *, *P* < 0.05; **, *P* < 0.01; ***, *P* < 0.001.

To determine whether a relationship exists between CRISPR and CPS in regulating bacterial physiology, we constructed nonpolar mutants with deletion of the *cpsE* gene, which encodes a glycosyltransferase that initiates synthesis of the polysaccharide repeating unit in type Ia GBS (24) in both the WT and ΔCRISPR backgrounds and in corresponding complement strains. Considering that the complemented CΔ*cpsE* and ΔCRISPR CΔ*cpsE* strains have a spectinomycin resistance (Spc^r^) plasmid present, we transformed the empty pSET2 plasmid into the WT and knockout strains so that all the strains were all exposed to the same antibiotic. Transmission electron microscopy illustrated that inactivation of *cpsE* resulted in an acapsular phenotype in the Δ*cpsE* and ΔCRISPR Δ*cpsE* strains, while the other strains, including those without deletion or complementation after deletion, exhibited an encapsulated phenotype (Fig. 2A). Furthermore, we assayed the content of sialic acid in the bacterial strains described above. As shown in Fig. 2B, Δ*cpsE* and ΔCRISPR Δ*cpsE* strains exhibited decreased sialic acid levels compared to the WT strain, while the complementation of *cpsE* in these two deletion mutants resulted in the recovery of sialic acid levels to large extents. No significant difference was observed in sialic acid production between the Δ*cpsE* and ΔCRISPR Δ*cpsE* mutants (Fig. 2B). To determine whether alteration in capsular thickness is associated with the CPS shedding, we measured the content of sialic acid in the supernatant of S. agalactiae strains. We could not detect any sialic acid in the supernatant of ΔCRISPR or WT strains (data not shown), indicating that capsular alteration is due to CPS synthesis rather than the release of capsular material into the medium. Then we investigated the surface physicochemical properties of individual bacterial strains. Compared to the WT strain, the ΔCRISPR mutant showed a significant decrease in autoaggregation rate (Fig. 2C) and surface hydrophobicity (Fig. 2D) and an increase in anionic charge (Fig. 2E), while loss of *cpsE* in the ΔCRISPR strain caused the elevation of the above parameters to levels comparable to those in the Δ*cpsE* mutant. These results indicated that CRISPR positively affected autoaggregation, surface hydrophobicity, and anionic charge by repressing CPS production.

### CRISPR promotes adhesion, invasion, and biofilm formation by inhibiting CPS production.

**FIG 3:**
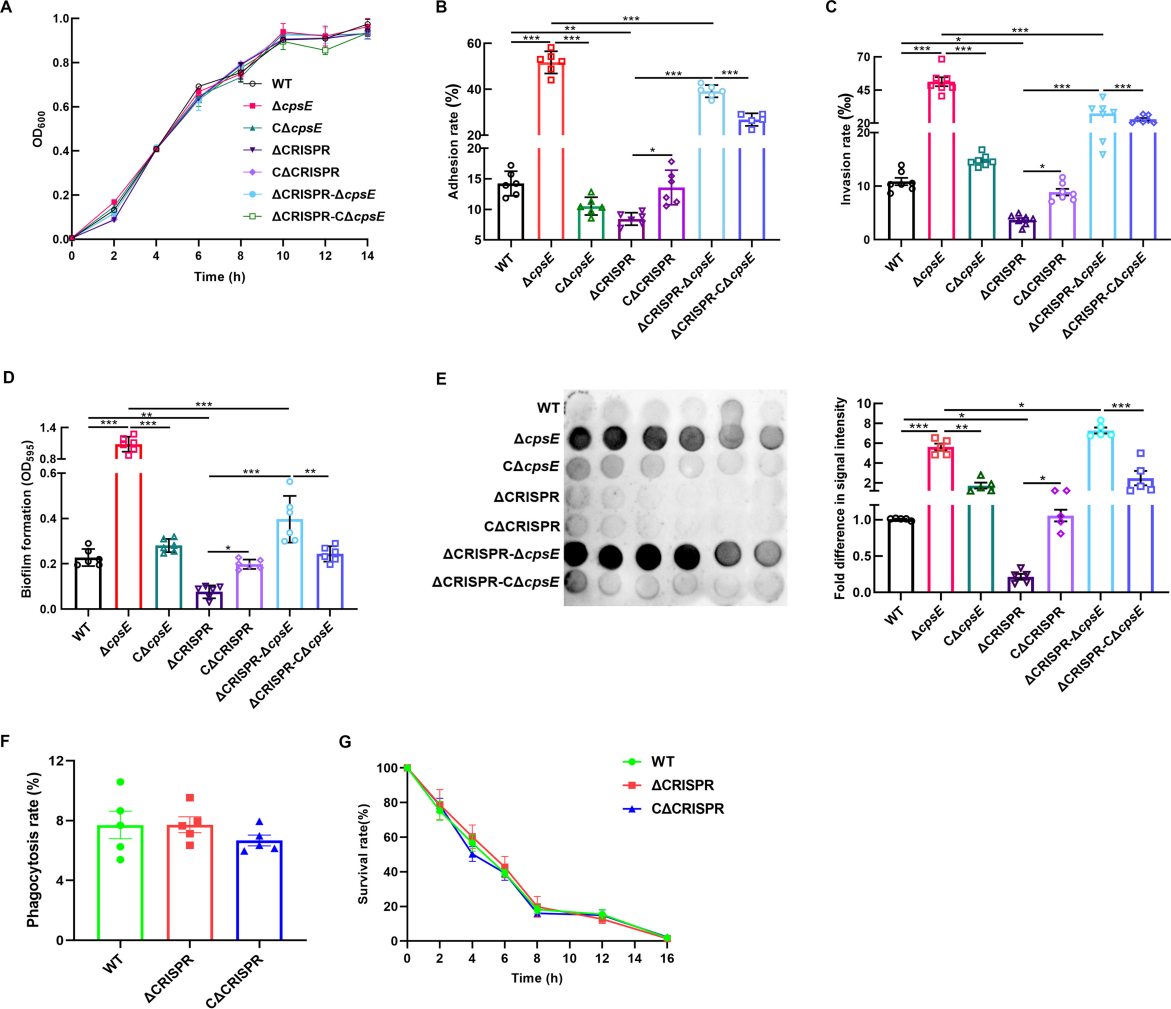
CRISPR promoted bacterial adhesion, invasion and biofilm formation by inhibiting CPS production. (A) Growth curves of the WT, Δ*cpsE*, CΔ*cpsE*, ΔCRISPR, CΔCRISPR, ΔCRISPR Δ*cpsE*, and ΔCRISPR CΔ*cpsE* strains. The S. agalactiae strain was grown in THB at 37°C, and the OD_600_ was measured every hour. (B) Adhesion ability of the indicated strains to bEnd.3 cells; (C) invasion ability of the indicated strains with respect to bEnd.3 cells. Bacteria were incubated with the cells for 2 h at 37°C. For the adhesion assay, the cells were washed and lysed to measure the number of CFU. For the invasion assay, the cells were treated with antibiotics to remove extracellular bacteria and then washed and lysed to measure the number of CFU. (D) Biofilm formation of the indicated strains. Biofilm formation was measured by crystal violet staining at OD_595_. (E) Dot blot analysis of the indicated strains. Cells were grown in THB medium and harvested in stationary phase. Whole-cell extracts were diluted as described in Materials and Methods, and dilutions were subjected to dot blot analysis using hyperimmune serum against S. agalactiae GD201008-001. (F) Phagocytosis rate of the WT and ΔCRISPR strains in RAW264.7 macrophages. (G) Intracellular survival of the WT and ΔCRISPR strains in RAW264.7 macrophages. The data are shown as the means ± SEM from three independent experiments. *, *P* < 0.05; **, *P* < 0.01; ***, *P* < 0.001.

Considering that bacterial surface properties may affect some virulence-associated phenotypes, such as adhesion, invasion, and biofilm formation, it was of interest to determine whether relationships exist among CRISPR, CPS, and these phenotypes. Before that, we examined the growth differences among the strains. The growth curves revealed no significant difference in growth rate between the WT strain and its derivative mutant strains (Fig. 3A). Then, we compared the adhesion and invasion rates of the S. agalactiae strains with respect to bEnd.3 brain microvascular endothelial cells. As shown in Fig. 3B and C, both the adhesion and invasion abilities of ΔCRISPR were reduced compared with those of the WT strain, while the absence of *cpsE* in ΔCRISPR caused significantly enhanced adhesion and invasion levels. The adhesion and invasion abilities of the Δ*cpsE* and ΔCRISPR Δ*cpsE* mutants were partly restored to WT or ΔCRISPR levels after *cpsE* complementation. Consistent with the above observations of the two phenotypes, the biofilm production of the ΔCRISPR strain was decreased compared to that of the WT strain, while the absence o*f cpsE* in the ΔCRISPR strain caused biofilm formation to be repressed 2-fold compared with that of the WT strain (Fig. 3D). Dot blot analysis with rabbit hyperimmune serum against S. agalactiae whole cells indicated that the signal intensity was increased in the Δ*cpsE* and ΔCRISPR Δ*cpsE* strains compared with those in the WT and ΔCRISPR strains (Fig. 3E). Intracellular growth of the WT and ΔCRISPR strains in RAW264.7 macrophages showed that the deletion of CRISPR had no effect on phagocytosis (Fig. 3F) and intracellular survival (Fig. 3G) of S. agalactiae.

### Upregulation of CPS is not associated with virulence attenuation in the ΔCRISPR mutant.

**FIG 4:**
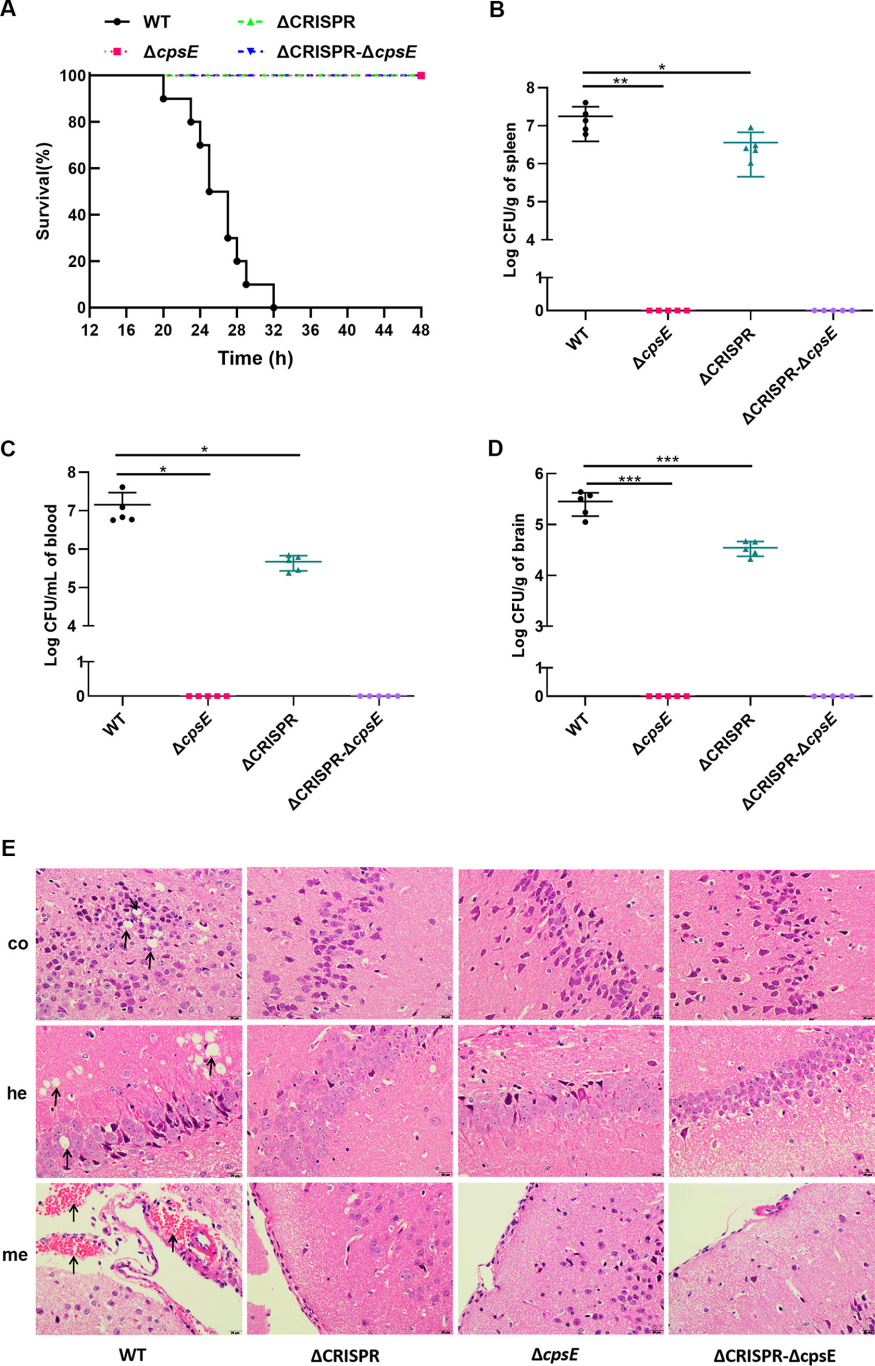
Upregulation of the *cps* operon is not associated with virulence attenuation in the ΔCRISPR mutant. (A) Survival percentages of mice infected with the WT, Δ*cpsE*, CΔ*cpsE*, ΔCRISPR, CΔCRISPR, ΔCRISPR Δ*cpsE*, and ΔCRISPR CΔ*cpsE* strains. (B to D) Bacterial loads in the spleens (B), blood (C), and brains (D) of mice infected with the indicated strains; (E) histopathological changes shown by H&E staining in brains from mice infected with S. agalactiae strains. me, meninges; hc, hippocampus; co, cortex. The data are shown as the means ± standard deviations (SD) from three independent experiments. *, *P* < 0.05; ***, *P* < 0.001.

To determine whether the attenuated virulence of the ΔCRISPR mutant was due to the upregulation of CPS, we investigated the mortality rates of the WT and its derived mutant strains in mice. Mice infected with the WT strain rapidly succumbed to disease and death, with 100% mortality occurring within 32 h. However, no death was observed in the mice infected with the Δ*cpsE* or ΔCRISPR Δ*cpsE* mutant (Fig. 4A). The analysis of bacterial burden indicated that at 20 h postinfection, the bacterial loads were up to 3.20 × 10^5^ CFU/g in the brain (Fig. 4B), 1.22 × 10^7^ CFU/mL in the blood (Fig. 4C), and 1.36 × 10^7^ CFU/g in the spleen (Fig. 4D) in mice infected with the WT strain. In mice infected with the ΔCRISPR mutant, bacterial loads were 3.36 × 10^4^ CFU/g in the brain, 5.16 × 10^5^ CFU/mL in the blood, and 2.56 × 10^6^ CFU/g in the spleen, respectively. No bacteria were detected in the brain, blood, or spleen of mice infected with the Δ*cpsE* or ΔCRISPR Δ*cpsE* strain. The virulence difference between the WT and ΔCRISPR strains for mice was further confirmed by histopathological observation. At 20 h postinfection, the brains of WT-infected mice showed prominent meningeal hemorrhage, erythrocyte aggregation, and accumulation of autophagic vacuoles in the cortex and hippocampus, whereas no obvious lesions were observed in the brains of mice challenged with the ΔCRISPR strains (Fig. 4E). All of these results suggested that virulence attenuation in the ΔCRISPR mutant could not be attributed to upregulated production of CPS.

### crRNA inhibits the expression of CPS by repressing the promoter activity of the *cps* operon.

**FIG 5:**
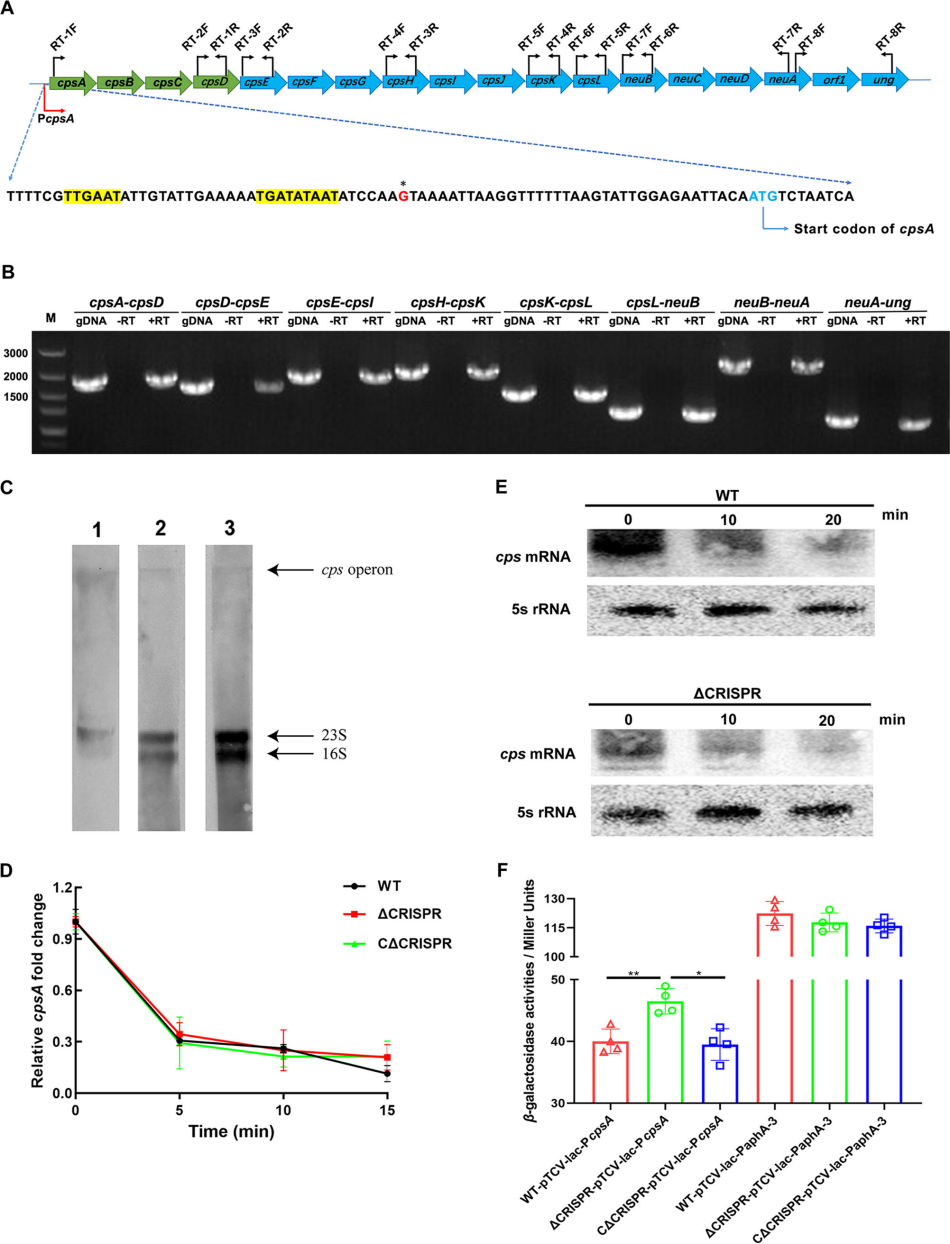
crRNA inhibits the expression of CPS by repressing promoter activity instead of mRNA cleavage of the *cps* operon. (A) Schematic representation of the *cps* operon. The black arrows represent primers designed for RT-PCR. The putative −10 and −35 boxes of promoters are highlighted in yellow. The transcription start sites are in boldface and marked with an asterisk. (B) The operon structure was analyzed by RT-PCR. RT amplification was performed with cDNA transcribed from total RNA as the template, genomic DNA (gDNA) amplification was performed with total chromosomal DNA as the template, and negative RT amplification was performed with mRNA as the template. (C) Northern blotting of the structure of the capsular operon of S. agalactiae. RNA was hybridized with *cpsA* (lane 1), *cpsJ* (lane 2), and *neuA* (lane 3) (D) Time course of *cpsA* stability in the indicated strains after treatment with rifampin; (E) Northern blot analyses of *cps* transcript in the WT and ΔCRISPR strains. Hybridization with 5S rRNA was used as an internal control. (F) β-Galactosidase activities of *lac-*P*cpsA*. The P*aphA-3*-*lacZ* integration vector containing the promoter region of *aphA-3* served as a negative control. The data are shown as the means ± SEM from three independent experiments. *, *P* < 0.05; **, *P* < 0.01.

By sequence analysis, we found that the CPS gene cluster in S. agalactiae GD201008-001 was located within a 17,060-bp DNA region harboring 18 complete open reading frames (ORFs), sequentially designated *cpsA*, *cpsB*, *cpsC*, *cpsD*, *cpsE*, *cpsF*, *cpsG*, *cpsH*, *cpsI*, *cpsJ*, *cpsK*, *cpsL*, *neuB*, *neuC*, *neuD*, *neuA*, *orf1*, and *ung* (Fig. 5A), which were named according to genome alignment with reference genomes of S. agalactiae OI1 (25). The potential −35 (TTGAAT) and −10 (GTTTAAACT) sequences were identified upstream of *cpsA* (Fig. 5A). RT-PCR analysis was used to dissect the structure of the CPS biosynthetic gene cluster. As shown in Fig. 5B, a product that corresponds to the transcript from the intergenic region from *cpsA* to *ung* could be obtained by RT-PCR amplification. This finding indicated that the CPS biosynthetic gene cluster was cotranscribed as an operon. To further confirm this, RNA was isolated and hybridized with oligonucleotide probes from various regions of the *cps* gene cluster. As shown in Fig. 5C, long transcripts (about 17 kb) were observed with *cpsA* (lane 1), *cpsJ* (lane 2), and *neuA* (lane 3) probes, which showed that the 18 genes constitute a single transcription unit. To map the transcription initiation site of this operon, 5′-rapid amplification of cDNA ends (5′-RACE) analysis was conducted using cDNA as the template. The transcription initiation site was shown in Fig. 5A. A noticeable phenomenon is that the extent of the amplification of the intergenic region between *cpsD* and *cpsE* was far less than that of other intergenic regions upon cotranscription of CPS gene cluster. Similar result has been found in S. agalactiae OI1, which was explained by the possible partial cessation of transcription between the 3′ end of *cpsD* and the 5′ end of *cpsE* (25).

To determine whether crRNA-mediated suppression of CPS synthesis was due to the cleavage of the target transcript, an RNA degradation assay was performed. Unexpectedly, no significant difference was observed in *cps* mRNA stability between the WT and ΔCRISPR strains (Fig. 5D). Northern blot analysis also demonstrated that the mRNA degradation rate of the capsular operon of the ΔCRISPR mutant was similar to that of the WT strain (Fig. 5E). These data indicated that the repression of CPS expression by crRNA was not due to the base pairing of the crRNA with the *cps* mRNA, which resulted in degradation of the target transcript. We speculated that crRNA may repress the transcription of *cps* by binding to its promoter. To explore whether crRNA could repress the transcription of the *cps* operon, we constructed a P*cps*-*lacZ* fusion containing a 320-bp fragment (bp 1188185 to 1188504) of the *cpsA* promoter and detected the promoter activity of the *cps* operon in the WT and ΔCRISPR strains by β-galactosidase activity assay. A P*aphA-3*-*lacZ* fusion plasmid containing a promoter that was not regulated by CRISPR served as a negative control. The results showed that P*cpsA* activity was increased in the ΔCRISPR mutant compared to the WT strain, whereas the promoter activity of P*aphA-3* was not affected by CRISPR deletion (Fig. 5F).

## DISCUSSION

The CRISPR-Cas system functions classically as a prokaryotic defense mechanism against invading phages and plasmids (26). Additional roles of this system in regulating bacterial endogenous genes are gradually being unraveled (6, 27). Among 125 S. agalactiae strains with published whole-genome sequences, 80 strains were identified to harbor the CRISPR arrays, except for strain GD201008-001, used in this study (see Table S1 in the supplemental), indicating that CRISPR is widely distributed in S. agalactiae genomes. In our previous study, the deletion of CRISPR in strain GD201008-001 created a large number of transcriptionally altered genes, among which several CPS genes were significantly upregulated (22). To support this transcriptomic finding, in the present study, we assayed the production of CPS and obtained evidence that CRISPR negatively affected the synthesis of CPS. The capsule is a widely recognized virulence determinant that protects bacteria from phagocytosis (28). Our previous and current data showed the ΔCRISPR mutant was highly attenuated but exhibited enhanced CPS production, which seems to be a contradiction. In fact, despite the importance of the capsule to the virulence of GBS, acapsular strains have clinically been isolated, which suggests that loss of the capsule may benefit certain aspects of GBS pathogenesis (29). Therefore, it is of interest to characterize the regulatory role of CRISPR RNA in CPS production and its contribution to S. agalactiae infection.

Considering that the capsule represents the outermost layer of the GBS cell wall, we hypothesized that loss of CPS might affect bacterial surface physicochemical properties, thereby altering some virulence-associated phenotypes. To test this hypothesis, we constructed an acapsular phenotype of S. agalactiae by inactivating the *cpsE* gene. As expected, the Δ*cpsE* mutant displayed increased autoaggregation and surface hydrophobicity and decreased anionic charge. This finding is in agreement with some previous studies that indicated that sialic acid is negatively correlated with surface hydrophobicity and autoaggregation (30, 31). Furthermore, we disrupted *cpsE* in the ΔCRISPR background to determine the relationship between the regulatory role of CRISPR in CPS production and the altered surface properties. As a result, all the physicochemical properties associated with CPS were regulated by CRISPR. Bacterial hydrophobicity and autoaggregation are important for colonization and biofilm development in flowing environments (32). Additionally, significant correlations among adhesion, hydrophobicity, and low electronegativity have been observed (33). Consistent with the observations of surface physicochemical properties, we found that loss of CPS improved the bacterial abilities of adhesion, invasion, and biofilm formation. In contrast to the ΔCRISPR mutant, which exhibited significantly decreased adhesion, invasion, and biofilm formation abilities, the ΔCRISPR Δ*cpsE* double mutant strain exhibited restored phenotypes. These findings suggest that the altered phenotypes observed in the ΔCRISPR mutant are linked to the upregulation of *cpsE*. However, notably, this upregulation of CPS may not be entirely responsible for the altered phenotypes (adhesion, invasion, and biofilm formation) observed in the ΔCRISPR mutant since the ΔCRISPR Δ*cpsE* mutant exhibited a more pronounced alteration than the WT strain. Additionally, compared with the Δ*cpsE* strain, the ΔCRISPR Δ*cpsE* strain exhibited weaker adhesion, invasion, and biofilm formation abilities, implying that the deletion of CRISPR can also affect the above abilities in an unknown way, in addition to upregulating the CPS expression.

Bacterial surface proteins play a vital role in host-microbe interactions (34), but not all of them can reach beyond the capsular layer. Schembri et al. (35) demonstrated that the capsule blocks antigen 43 through physical shielding in Escherichia coli. In Streptococcus pyogenes, the capsule masks biofilm-associated surface adhesins, such as M protein (36). Therefore, we speculate that loss of the CPS might result in the exposure of surface proteins and thus alter the above surface-associated phenotypes. This idea is supported by the data from the dot blot analysis with the hyperimmune serum against GD201008-001 whole cells, which revealed decreased signal intensity in the ΔCRISPR strain and the restoration of signal intensity after the deletion of *cpsE* in the ΔCRISPR strain. Similar phenomena have been reported in a previous study on Neisseria lactamica (37), in which the hyperimmune serum to whole bacterial cells was verified to exhibit strong reactivity to surface proteins. Different from most of the bacterial capsules, the sialic acid capsule of GBS is nonimmunogenic and does not act as an antigen. It has been reported that the capsular sialic acid of GBS is similar to mammalian sialic acid, which can be recognized by mammalian sialic acid recognizing immunoglobulin-like receptors (Siglecs), allowing GBS to masquerade as “self” and thereby elude host immune responses (15).

Expression of CPS is essential for systemic virulence because of its antiphagocytic properties, which has been well characterized in S. agalactiae (38). To investigate whether increased CPS expression affects the antiphagocytosis and survival abilities of the ΔCRISPR mutant in macrophages, we performed phagocytosis assays in RAW264.7 macrophages. Surprisingly, the deletion of CRISPR had no effect on phagocytosis and intracellular survival of S. agalactiae, indicating that capsule may not be the only factor for this bacterium to resist phagocytosis. Also, this finding probably shows the fact that the attenuation mechanism of the ΔCRISPR strain is not fully associated with macrophage defense. It is certainly worth noting that the *in vitro* observations in bacterial antiphagocytosis activity might be inconsistent with the evaluation *in vivo*. Furthermore, our data indicated that enhanced CPS was not responsible for the attenuated phenotype of the ΔCRISPR strain because if the virulence attenuation of the ΔCRISPR strain results from enhanced CPS levels, then inactivation of the *cpsE* gene should rescue ΔCRISPR. A previous study on Streptococcus suis indicated that small RNA rss04 represses CPS production but contributes to the induction of meningitis in mice (39), which is in line with our finding. Although our data on mice suggests that CPS is a very important virulence factor of S. agalactiae, it does not invalidate our speculation that this bacterium may dynamically adjust CPS production at different stages of infection in order to survival in different niches. At an early colonization stage, Streptococcus pneumoniae reduced the levels of capsule in order to expose underlying surface molecules that facilitate adherence to the host epithelial cells and promote biofilm formation (40–42). Conversely, during a systemic bacterial infection, enhanced levels of capsule are necessary to resist destruction mediated by complement-mediated opsonophagocytosis (43, 44). Therefore, we hypothesize that the repression of CRISPR on capsule production might be beneficial for S. agalactiae invasion in the early stage of infection. This idea is supported by our data that the WT strain has the higher ability to invade mouse brain compared with the ΔCRISPR mutant strain.

Notably, our study showed that all the mice infected with the ΔCRISPR mutant survived, but the bacterial loads in the tissues, especially the brain, were not as low as expected at 20 h postinfection. Actually, this result is not surprising. Our previous study indicated that deletion of CRISPR caused enhanced expression of Toll-like receptor 2 (TLR2)-activating lipoprotein Sag0671 and thus increased the innate immune response (23). It is known that glial cells, including microglia, astrocytes, and oligodendrocytes, are the primary cellular components of the central nervous system (CNS) and involved in the immune response in a variety of ways (45). Therefore, we speculate that the ΔCRISPR mutant was likely to be eliminated by physiological inflammation and subsequent immune reactions in the brain tissue. Analysis of the dynamic bacterial loads in the brains of mice infected with the ΔCRISPR strain showed that from 24 h postinfection, the ΔCRISPR strain was initially largely cleared from the brain and almost undetectable by 108 h postinfection (Fig. S1). This finding may reflect a role of host immune factors. The attenuation mechanism of the ΔCRISPR mutant is quite complicated, since our recent study demonstrated that CRISPR deprivation influenced a large number of genes involved in diverse physiological processes (23). The molecular mechanisms by which CRISPR governs the virulence of S. agalactiae remain to be clarified.

In some bacteria, the role of CRISPR-Cas in controlling endogenous gene expression has been demonstrated to involve an RNA-based mechanism. For example, in Campylobacter jejuni, crRNA can guide Cas9 to bind and cleave complementary endogenous mRNAs (46). Our previous study indicated that *cas9* could mediate the degradation of *regR* mRNA in S. agalactiae GD201008-001 (22). In addition, our recent transcriptome combined with Freiburg RNA platform analysis showed that a large number of upregulated genes in the S. agalactiae ΔCRISPR strain, which included three genes (*cpsB*, *cpsC*, and *cpsJ*) of the *cps* operon, were partly hybridized with one or more CRISPR spacers (23). These findings led us to suspect that crRNA may mediate the repression of the *cps* operon via the degradation of crRNA-mRNA pairs. Unexpectedly, deletion of crRNA did not alter the degradation rate of *cps* transcripts following treatment with rifampin, suggesting that crRNA-mediated regulation was not dependent on direct interaction with the mRNA of the *cps* operon. It has been reported that noncanonical small RNA (scaRNA) can partially base pair with the promoter of endogenous genes in Francisella novicida, thus targeting Cas9 to repress transcription of target genes (47). To identify the mechanism by which crRNA regulates CPS production, we detected the promoter activity of the *cps* operon in the WT and ΔCRISPR strains by β-galactosidase activity assay. The result showed that crRNA could inhibit the promoter activity of *cpsA*, a putative transcriptional regulator of the capsule locus. Therefore, we speculate that the deletion of CRISPR may impact the targeting of Cas9 to the promoter of *cpsA* and thereby release the transcriptional inhibition of the *cps* cluster. Although we cannot rule out the possibility of an alternative mechanism of CRISPR-mediated gene regulation, the data presented here clearly demonstrate that transcriptional interference is the dominant contributor.

Taken together, the results of our study indicate that CRISPR inhibits the transcription of the *cps* operon by repressing the activity of promoter P*cpsA*, leading to increases in the abilities of adhesion, invasion, and biofilm formation in S. agalactiae. The findings presented here expand our understanding of CRISPR functions in modulating bacterial physiology.

## MATERIALS AND METHODS

### Bacterial strains, plasmids, and growth conditions.

S. agalactiae strain GD201008-001 (β-hemolysin or cytolysin positive), which belongs to serotype Iα and multilocus sequence type (MLST) ST7, was isolated from tilapia with meningoencephalitis in the Guangdong Province of China in 2010 (21). The bacterial strains and plasmids used in this study are listed in Table 1. All S. agalactiae strains were grown in Todd-Hewitt broth (THB) or plated on THB medium with 1.5% (wt/vol) agar. Escherichia coli strain DH5α was used as the host for plasmids and was cultured in Luria-Bertani (LB) broth or on LB agar medium. When necessary, antibiotics were used as follows: 100 μg/mL spectinomycin (Spc) or 10 μg/mL erythromycin (Ery) for S. agalactiae and 50 μg/mL Spc or 50 μg/mL kanamycin (Kan) for E. coli.

**TABLE 1:** Bacterial strains and plasmids

Strain or plasmid	Description	Source or reference
Strains		
GD201008-001	Serotype Ia, ST-7, tilapia clinical isolate	21
ΔCRISPR mutant	CRISPR locus deletion mutation in GD201008-001	23
CΔCRISPR complement	CRISPR locus complementation of strain in genome original position	23
Δ*cpsE* mutant	*cpsE* deletion mutation in GD201008-001	This study
CΔ*cpsE* complement	Δ*cpsE* mutant with plasmid pSET2-*cpsE*	This study
ΔCRISPR Δ*cpsE* mutant	In-frame deletion of CRISPR loci and *cpsE* in GD201008-001	This study
ΔCRISPR C*cpsE* complement	ΔcrRNA Δ*cpsE* with plasmid pSET2-*cpsE*	This study
DH5α	F^−^ Δ(*lacZYA-argF*)*U169 recA1 endA1 hsdR17*(r_K_^−^, m_K_^+^) *phoA supE44* λ^−^	22
Plasmids		
pSET4S	Spc^+^, thermosensitive suicide vectors	23
pSET2	Spc^+^, shuttle cloning vectors	23
pTCV-lac	Erm^+^ Kan^+^ promoter-*lacZ* fusion vector	56

### Construction of the mutant and complement strains.

The ΔCRISPR mutant strain and the CΔCRISPR complement strain were constructed in our previous study (23). The deleted targets (bp 941238 to 941802) encoded in the CRISPR array are shown in Fig. S2 in the supplemental material. The *cpsE* gene deletion mutant strain was generated using homologous recombination, as described previously (22). The left and right arms of the *cpsE* gene were amplified using the primer pairs cpsE-P1/cpsE-P2 and cpsE-P3/cpsE-P4, and the arms were then used as the templates to generate fusion fragments with the primer pair cpsE-P1/cpsE-P4. All primers are listed in Table 2. The fusion fragments were cloned into the pSET4s, which was digested by the restriction enzyme EcoRI (TaKaRa, China) to generate the *cpsE* deletion vector pSET4s-*cpsE* in E. coli DH5α. The constructed plasmids were verified by sequencing. The recombinant plasmid pSET4s-*cpsE* was transformed into S. agalactiae GD201008-001 competent cells by electroporation at 2.35 kV, 200 Ω, and 25 μF using the GenePulser Xcell electroporation system (Bio-Rad, USA). After being incubated for 3 h at 28°C in THB supplemented with 0.2% yeast extract and 30 mM sucrose without antibiotics to allow cell recovery, the transformed cells were plated on Todd-Hewitt (TH) agar medium with 100 μg/mL Spc and incubated at 28°C for 12 to 24 h. Because the thermosensitive suicide vector pSET4s cannot replicate by itself in bacterial cells at 37°C, the single-crossover mutant, in which pSET4s-*cpsE* was integrated into the upstream or downstream region of the chromosomal *cpsE* gene, was obtained by shifting the incubation temperature of the transformants to 37°C with 100 μg/mL Spc. The single-crossover mutant obtained was then subcultured for at least 6 passages in THB at 28°C without antibiotics. During the subculture, a second set of recombination events may happen, and so the single-crossover mutant was expected to become a Δ*cpsE* mutant or return to wild type. The subculture cells were plated on TH agar medium without antibiotics at 37°C overnight to select the Δ*cpsE* mutant. Colonies that formed on the plates were inoculated onto TH agar medium with and without 100 μg/mL Spc and cultured at 37°C overnight. Spc-susceptible colonies, which may have lost the target *cpsE* gene or returned to wild type, were selected. The Δ*cpsE* mutant was distinguished from the WT strain by PCR using the primers cpsE-P1/cpsE-P4 and cpsE-A/cpsE-B and verified by DNA sequencing. The ΔCRISPR Δ*cpsE* strain was constructed in the ΔCRISPR background using the same approach.

**TABLE 2:** Primers used for PCR amplification

Primer	Sequence (5′→3′)	Function
cpsE-P1	AAAACGACGGCCAGTGAATTCTGCTACAGCGGCACCAG	Construction of *cpsE* deletion mutant
cpsE-P2	GCTCTTGTATAAGGTAAGGTTTGAAAGGAAT	
cpsE-P3	ACCTTACCTTATACAAGAGCCCCTTACTTCCTT	
cpsE-P4	CCGGGTACCGAGCTCGAATTCTACCACCAAATCCAACAGCAT	

cpsE-A	TCATCAACTGTGGGAGGG	Verification of *cpsE* deletion mutant
cpsE-B	TTGAAGCAATGGGAGTGA	

CcpsE-F	GAGCTCGGTACCCGGGGATCCTCCAACAGCATTACTTCAGA	Construction of *cpsE* complement strain
CcpsE-R	CAGGTCGACTCTAGAGGATCCGGCACCAGATGATATGATAAC	

RT-1F	CAGGTTTGTATGGCGTTGAG	Cotranscription analysis of *cpsA-cpsD*
RT-1R	TGCTGTTGGATTTGGTGGT	
RT-2F	CTCGTTTAGAAATAGTTGATA	Cotranscription analysis of *cpsD-cpsE*
RT-2R	ACCTTTAGTGTTAGGAGAATA	
RT-3F	GAACACGCCCTCCCACAG	Cotranscription analysis of *cpsE-cpsI*
RT-3R	ATGAGTCTGTTGTAGTACGGATTG	
RT-4F	TTGATTGGGTCAGACTGGATT	Cotranscription analysis of *cpsH-cpsK*
RT-4R	ATTCGTGGTCACTCACATTACA	
RT-5F	TGTAATGTGAGTGACCACGAAT	Cotranscription analysis of *cpsK-cpsL*
RT-5R	CCAAGCGAATAACCGAAAC	
RT-6F	GGGTCAAATACGGAGAAGAAT	Cotranscription analysis of *cpsL-neuB*
RT-6R	CCACAAGACACCGCAACAT	
RT-7F	AATGGTAGATGTTGCGGTGTC	Cotranscription analysis of *neuB-neuA*
RT-7R	CGCCGTTCGGATAGTATAAAG	
RT-8F	AATCAATCAATGGCTAACAG	Cotranscription analysis of *neuA-ung*
RT-8R	TTGAAGTGAAGGAGGAGC	

recA-F	ATTCAGGCGCAGTTGATTTAGTT	qRT-PCR for *recA*
recA-R	TCAATCTCAGCACGAGGAACA	
q-cpsA-F	GCAGGCTTTGGGCTTTGTTAG	qRT-PCR for *cpsA*
q-cpsA-R	GGACGTTTGGAGCTGTGAGGT	
q-cpsB-F	TTAGCACGTTCTTTACCGAAATCT	qRT-PCR for *cpsB*
q-cpsB-R	CCTTGATGTTAGACCGCCATT	
q-cpsC-F	GCAACAACTGAGACGAAGGATAA	qRT-PCR for *cpsC*
q-cpsC-R	ACGCCACAATATACTTCTTCAACA	
q-cpsE-F	TACAACGACACGACTTTC	qRT-PCR for *cpsE*
q-cpsE-R	ATCATAACAATCCTTTTCAG	
q-cpsJ-F	TCGCTATGTAATCAGAGGAAACTT	qRT-PCR for *cpsJ*
q-cpsJ-R	CGAATGGTGGGTTGTCAGAA	
q-neuA-F	CCAAATTGCCGGTAGAGCTATT	qRT-PCR for *neuA*
q-neuA-R	CGCAGTGGACGAATGTGTTAT	

Pro-cpsA-F	CAAATGAATTCCCGGGGATCCCCCATCTCAGTCTCAAGGTTC	Promoter of *cpsA* fused to pTCV-lac
Pro-cpsA-R	GTATCAACAAGCTGGGGATCCTGTAATTCTCCAATACTTAAAAAA	

Pro-aphA-3-F	CAAATGAATTCCCGGGGATCCCCCAGCGAACCATTTGAGG	Promoter of *aphA-3* fused to pTCV-lac
Pro-aphA-3-R	GTATCAACAAGCTGGGGATCCCCGATTTTGAAACCAC	

5′RACE-GSP	CGGACGTTTGGAGCTGTGAGGTAATGCTAT	5′-RACE assay
5′RACE-NGSP	CTCTAACAAAGCCCAAAGCCTGCTCAGA	

To generate the corresponding complement strain, we constructed the plasmid pSET2-*cpsE*, which contains an open reading frame (ORF) of *cpsE*. Briefly, the fragment containing the ORF of *cpsE* was amplified using the primers CcpsE-F/CcpsE-R and cloned into the shuttle cloning vectors pSET2, which were digested by the restriction enzyme BamHI (TaKaRa, China). As mentioned above, the recombinant plasmid pSET2-*cpsE* was electroporated into the Δ*cpsE* mutant and screened on TH agar medium with 100 μg/mL Spc.

The positive clones were verified by PCR. The ΔCRISPR CΔ*cpsE* complement strain was constructed by the same approach, except that ΔCRISPR Δ*cpsE* strain was used as the parent strain. The schematic diagram of the construction of the mutant and complement strains is shown in Fig. S3.

### *In vitro* growth kinetics assay.

In order to maintain the same growth conditions as the complemented strains, we transformed the empty pSET2 plasmid into the WT and mutant strains. Overnight S. agalactiae strains were prepared and adjusted to an optical density at 600 nm (OD_600_) of approximately 0.6 with fresh THB medium. Cells were pelleted by centrifugation at 5,000 × *g* for 10 min, washed three times, and later resuspended in phosphate-buffered saline (PBS). Then, 300-μL aliquots of the suspension were inoculated into Erlenmeyer flasks containing 30 mL of fresh THB medium with Spc. Next, the Erlenmeyer flasks were incubated at 37°C with shaking. Bacterial growth was examined at regular intervals by monitoring the OD_600_ using a spectrophotometer (Bio-Rad, USA).

### RNA isolation, RT-PCR, and qRT-PCR.

The total RNA of bacterial cells was extracted as described previously (48). Briefly, bacteria were grown to mid-log phase in THB medium with Spc and then pelleted by centrifugation at 4°C for 10 min, resuspended in 800 μL lysis solution (2.7 g sodium acetate, 5 g SDS, and EDTA at 0.34 g/L deionized water [pH 5.5]) and disrupted using Lysing matrix B (MP Biomedicals, CA, USA). The supernatant was harvested into a new tube by centrifugation at 10,000 × *g* for 3 min and mixed with the same volume of saturated phenol (pH 5.5). The mixture was incubated at 68°C for 5 min and the phase was separated by centrifugation at 13,000 × *g* for 3 min. The aqueous layer was pipetted into a new tube and mixed with a double volume of a 30:1 mixture of ethyl alcohol (EtOH) to 3 M NaOAc (pH 6.5). Then, the mixture was incubated for 4 h at −80°C and centrifuged at 13,000 × *g* for 30 min, and the supernatant was carefully removed. The pellet was washed with 200 μL of 75% ethanol (vol/vol), stored at −20°C and centrifuged at 13,000 × *g* for 10 min, and the supernatant was carefully removed. After being air dried, 50 μL distilled water was added to the pellet, and the RNA was resuspended by shaking at 65°C for 5 min. Then, the RNA was synthesized into cDNA using HiScript II Q RT supermix (Vazyme, Nanjing, China). The primers used in RT-PCR and qRT-PCR are listed in Table 2. The level of mRNA expression was measured by two-step relative qRT-PCR. The *recA* gene was used as the internal control gene (49). SYBR green PCR was performed using AceQ qPCR SYBR green master mix (Vazyme, Nanjing, China) and the StepOnePlus real-time PCR system (Applied Biosystems, Waltham, MA, USA). Changes in gene transcription were determined using the comparative cycle threshold (2^−ΔΔ^*^CT^*) method (50).

### Transmission electron microscope.

Overnight cultures of bacteria were subcultured 1:100 to mid-log phase. The bacterial suspension was prepared by centrifugation at 1500 rpm for 10 min and fixed in 2.5% glutaraldehyde for more than 2 h. The samples were dehydrated in propylene oxide for 10 min, embedded in epoxy resin, and processed to a trapezoid shape having a surface area of less than 0.2 mm by 0.2 mm. Ultramicrotomy was performed on the embedded material to obtain a thickness of 50 to 90 nm. Subsequently, the thin sections were mounted onto 300 mesh copper grids, stained with alcoholic uranyl acetate and alkaline lead citrate, and washed with distilled water. The samples were observed on a Hitachi 600 transmission electron microscope.

### Quantification of CPS.

CPS was extracted as described previously (20). Briefly, bacteria were grown to mid-log phase in 30 mL THB at 37°C. The cell densities were measured based on OD_600_. Then, the cells were pelleted by centrifugation at 3,500 × *g* for 15 min, washed twice with PBS, suspended in 0.8 M NaOH, and incubated for 48 h at 37°C. After being neutralized with HCl, the samples were centrifuged at 10,000 × *g* for 10 min and filtered to remove the insoluble material. The supernatant was concentrated using Amicon Ultra10 filters (Millipore, Bedford, MA, USA). After being washed twice with distilled water, the extracted polysaccharide was collected from the membrane by resuspension in water. The concentrations of sialic acid were determined by a sialic acid assay kit (Jiancheng, Nanjing, China) according to the manufacturer’s instructions.

### Assessment of autoaggregation, hydrophobicity, and surface charge.

Overnight cultures were washed twice with PBS and adjusted to an OD_600_ of 1.0. Then, 1.5 mL of the bacterial suspension was pipetted into a 2-mL tube and incubated at 37°C for 2 h after being fully vortexed. The autoaggregation ability was determined by measuring the optical density of the culture supernatant at 600 nm (*A*_120_) and calculating the autoaggregation rate according to the following formula: autoaggregation rate = [(1.0 − *A*_120_)/1.0] × 100%.

The hydrophobic properties were evaluated by the microbial adhesion to hydrocarbons (MATH) method (51). The overnight cultures were inoculated into fresh THB medium at a ratio of 1:100 and grown to mid-log phase. The cells were pelleted by centrifugation, washed twice with PBS, and adjusted to an OD_600_ of 0.6. One milliliter of dimethylbenzene was mixed with 3 mL of bacterial suspension by vortexing for 100 s. The samples were incubated at 37°C for 40 min, and the optical density of the aqueous layer at 600 nm was measured as *A*_40_. Hydrophobicity was determined using the following formula: hydrophobicity = [(0.6 − *A*_40_)/*A*_40_] × 100%.

The anionic cell surface charge was measured by the cytochrome *c* assay (52). Briefly, the overnight cultures were diluted 1:100 in fresh THB and grown to mid-log phase. The cells were pelleted by centrifugation, washed twice with 3-morpholinopropane-1-sulfonic acid (MOPS) buffer (pH 7.0), and adjusted to an OD_600_ of 0.6. Fifty microliters of cytochrome *c* buffer (10 mg/mL) was mixed with 450 μL of bacterial suspension by pipetting, and the mixture was incubated at room temperature for 15 min. The bacteria were pelleted by centrifugation at 13,000 × *g* for 15 min, and the amount of unbound cytochrome *c* was determined by measuring the absorbance of the supernatant at 530 nm.

### Adherence assay.

The adherence assay was performed as previously described (53). bEnd.3 brain microvascular endothelial cells were cultured to 90% confluence in Dulbecco’s modified Eagle’s medium (DMEM; Gibco, New York, NY, USA) supplemented with 15% fetal bovine serum (FBS) (Gibco) in 24-well tissue culture plates. S. agalactiae *s*trains were grown to the mid-log phase in THB at 37°C, harvested by centrifugation at 5,000 × *g* for 10 min, washed three times in sterile 10 mM PBS, and resuspended in fresh serum-free DMEM. The monolayers were washed with PBS, and S. agalactiae was seeded into each well at a multiplicity of infection (MOI) of 1:1. The plate was centrifuged at 600 × *g* for 10 min and incubated at 37°C with 5% CO_2_ for 2 h to allow for cell adhesion. Subsequently, nonadherent bacteria were removed by washing the wells with PBS five times. After washing, 0.02% Triton X-100 was added to each well to lyse the cells. The lysates were serially diluted in PBS and plated to count the CFU after overnight culture.

### Invasion assay.

bEnd.3 cells were cultured to 90% confluence in DMEM with 10% FBS at 37°C with 5% CO_2_. Bacterial and cell monolayers were processed in the same way as described for the adhesion assay. Cocultured cells were incubated at 37°C for 2 h. The extracellular bacteria were removed by washing the monolayers with PBS and replaced with DMEM containing100 μg/mL penicillin for 40 min at 37°C with 5% CO_2_. Next, the infected cells were washed three times with PBS. Triton X-100 at 0.02% was added to each well to lyse the cells. Bacterial numbers were enumerated via 10-fold serial dilution and spread onto THB agar plates.

### Biofilm formation assay.

Biofilm formation was evaluated by a crystal violet (CV) assay as previously described (54). Briefly, overnight cultures were adjusted to an OD_600_ of 1.0 with THB and diluted 1:20 in THB supplemented with 1% glucose. The bacterial suspension was added to 96-well polystyrene microtiter plates (200 μL/well), and wells that were filled with THB alone served as a negative control. The plates were incubated at 37°C without shaking for 18 h. Next, the contents of the wells were poured off, and the planktonic bacteria were removed by washing twice with PBS. After the samples were fixed with 200 μL of methanol for 15 min, the methanol was removed. After drying for 15 min, the wells were stained for 10 min with 200 μL of a 1% (wt/vol) solution of CV. The unbound dye was washed away with double-distilled water (ddH_2_O). Then, the bound dye was released from the stained cells with 95% ethanol. Biofilm formation was quantified by measuring the OD_595_ of the samples.

### *S. agalactiae* phagocytosis and intracellular survival assay.

RAW264.7 macrophages were grown in DMEM containing 10% FBS in 24-well tissue culture plates at a concentration of 4 × 10^5^ cells/well. S. agalactiae strains were grown in THB for 3 h at 37°C and then harvested by centrifugation at 5,000 × *g* for 10 min. The cell monolayers were infected at a multiplicity of infection (MOI) of 1:1 for 1 h at 37°C. Extracellular bacteria were removed by washing with PBS five times, refilling the wells with 5 μg/mL penicillin G-containing 1% FBS–DMEM, and incubating the plates at 37°C in 5% CO_2_ for 1 h. To measure the phagocytotic rate, the cells were washed and lysed with 0.02% Triton X-100. Lysates were serially diluted in PBS and plated on TH agar medium, and the number of CFU was counted after incubation at 37°C overnight. To measure the survival rate of intracellular bacteria, cell samples were taken 1 h after the interaction with antibiotics (time point 0). At the subsequent 2-h, 4-h, 6-h, 8-h, 12-h, and 16-h time points, the cells were washed and lysed to measure the number of CFU. The relative survival rate was calculated as follows: (CFU at a specific time point/CFU at time point 0) × 100.

### Dot blot assay.

Dot blot assays were performed as previously described, with some modifications (55). Briefly, overnight cultures were inoculated into 10 mL THB at a ratio of 1:100, washed twice with PBS, and adjusted to 1 × 10^10^ CFU/mL. Aliquots (5 μL) of bacteria serially diluted 2-fold were spotted onto nitrocellulose membranes. After being fixed with 70% ethanol for 5 min and air dried, the membrane was blocked with 5% (wt/vol) skim milk in PBST (PBS containing 0.05% Tween 20) at 4°C overnight. Then, the membrane was incubated with hyperimmune serum (1:500 dilution) against S. agalactiae GD201008-001 at 37°C for 1.5 h and washed three times with PBST. The membrane was incubated with horseradish peroxidase (HRP)-conjugated goat anti-rabbit IgG (1:10,000 dilution) at 37°C for 1.5 h and washed three times with PBST. Finally, the reaction was visualized with Omni ECL reagent (EpiZyme, Shanghai, China) under a ChemiDoc touch imaging system (Bio-Rad, Hercules, CA, USA).

### Murine infection.

Female ICR mice were purchased from the Experimental Animal Center of Yangzhou University. Overnight cultures were diluted 1:100 in fresh THB, grown to mid-log phase, and then washed three times in PBS. The treatment groups were infected with 100 CFU of the indicated strains per animal in a 100-μL suspension by intraperitoneal administration, and their survival was monitored for 48 h postinfection. Control mice were injected with sterile PBS. For the bacterial burden assay, groups of five mice were sacrificed at 20 h postinfection, the brain and spleen were harvested and weighed, blood samples were collected, and the brain, spleen, and blood were then homogenized in PBS. The homogenates were serially diluted in PBS and plated to count the CFU after overnight culture. Animal experiments were implemented according to animal welfare standards and were approved by the Ethical Committee for Animal Experiments of Nanjing Agricultural University, China [permit no. SYXK (SU)0.2017-0007]. All animal experiments were performed in compliance with the guidelines of the Animal Welfare Council of China.

### Histological assessment.

The mice were infected with 100 CFU of the S. agalactiae strains in a 100-μL suspension by intraperitoneal administration. At 20 h postinfection, the infected mice were euthanized, and the brains were collected and placed into 10% neutral buffered formalin. After fixation, the tissues were dehydrated and embedded in paraffin, and 4-mm sections were taken and stained with hematoxylin and eosin (H&E) for histological evaluation.

### RNA degradation assay.

RNA degradation assays were performed as previously described (5). Briefly, overnight cultures were diluted to an OD_600_ of 1.0 in 10 mL THB. Rifampin (Sigma, St. Louis, MO, USA) was added to a final concentration of 500 μg/mL to prevent mRNA production, and aliquots were taken for RNA extraction every 5 min. Relative transcript levels were determined by qRT-PCR as described above.

### 5′-RACE.

The 5′-RACE assay was performed to map the transcription initiation site of the capsular operon. Total RNA was isolated from S. agalactiae during the exponential phase. The 5′-RACE procedure was performed using the HiScript-TS 5′/3′-RACE kit (Vazyme, Nanjing, China) following the manufacturer’s instructions. Briefly, the total RNA (1 μg) of bacterial cells was treated with 5′ random primer and deoxynucleoside triphosphate (dNTP) mix and heating at 72°C for 3 min. Subsequently, the RNA was reverse transcribed with FS buffer, enzyme mix and TS oligonucleotide to cDNA. The cDNA was then used as a template in the first PCR with the universal primer and the specific primer 5′RACE-GSP (Table 2). Then, the 50-fold-diluted amplification product was used as a template in nested pCR with the nested primer and the gene-specific primer 5′RACE-NGSP. The resulting 5′-RACE products were cloned into pClone007 using the pClone007 blunt simple vector kit (Tsingke, Beijing, China) for sequencing. More than three independent positive clones were used for sequence verification.

### Northern blot analysis.

Overnight cultures were diluted to an OD_600_ of 1.0 in 10 mL THB. Rifampin (Sigma Louis, MO, USA) was added to a final concentration of 500 μg/mL to prevent mRNA production, and aliquots were taken for RNA extraction at 0, 10, and 20 min. Fifty micrograms of total RNA was separated by 15% denaturing polyacrylamide gel electrophoresis (PAGE), electroblotted onto a nylon membrane, and then cross-linked under a 1,200-mJ UV light. The RNA was then hybridized with a digoxigenin-labeled probe for 12 h, After hybridization, membranes were washed in 2× standard sodium citrate (SSC)–0.1% SDS at room temperature for 5 min, followed by being washed in 0.1× SSC–0.1% SDS at 65°C for 30 min. Membranes were equilibrated for 1 min in maleic acid containing 0.3% Tween 20 and blocked for 30 min. After incubation for 45 min with a 1:10,000 dilution of antidigoxigenin-conjugated alkaline phosphatase (Boehringer Mannheim), membranes were washed for 30 min in maleic acid containing 0.3% Tween 20 and equilibrated for 5 min in 0.1 M Tris-HCl–0.1 M NaCl. The hybridization signal was visualized by using the chemiluminescent substrate CSPD (Boehringer Mannheim).

### Determination of β-galactosidase activity.

The β-galactosidase activity was evaluated using the *lacZ* transcriptional fusion plasmid pTCV-lac, as previously described (56). The primers used for *lacZ* transcriptional fusions are listed in Table 2. Briefly, the putative promoter region was fused to the *lacZ* reporter gene and transformed into the WT, ΔCRISPR, and CΔCRISPR strains. The positive clones were selected by erythromycin resistance and verified by PCR. Then, overnight cultures were diluted 1:100 and grown to an OD_600_ of 1.0. A 20-mL bacterial suspension was pelleted by centrifugation at 5,000 × *g* for 10 min at 4°C, washed three times with PBS, and then resuspended in 200 μL β-mercaptoethanol (BME)-free Z buffer (60 mM Na_2_HPO_4_, 40 mM NaH_2_PO_4_, 10 mM KCl, 1 mM MgSO_4_·7H_2_O). The diluted cells were permeabilized by treatment with 0.05 M BME, 0.5% toluene, and 4.5% ethanol for 5 min at 30°C. The substrate *o*-nitrophenyl-β-d-galactopyranoside (ONPG; 4 mg/mL) was added to start the reaction until a sufficient yellow color developed. The β-galactosidase activity was expressed as follows: [10^3^ × (OD_420_ of the reaction mixture − 1.75 × OD_550_ of the reaction mixture)]/[time of the reaction (minute) × OD_600_ of the quantity of cells used in the assay]. An unrelated promoter that is not regulated was used as the negative control.

### Statistical analyses.

Statistical analyses were performed using GraphPad Prism (version 9.0.2) (GraphPad Software, La Jolla, CA, USA). One-way analysis of variance (ANOVA) was performed to analyze the data from the experiments for bacterial surface characteristics, biofilm formation, adherence, invasion, and qRT-PCR. The nonparametric Mann-Whitney U test was used to analyze the results of the animal infection study. Statistical significance was considered at a *P* value of *<*0.05.

### Data availability.

The RNA-seq data generated from this study were submitted to the NCBI under BioProject accession no. PRJNA413672 and PRJNA831578.
