# Supplementary material for: CRISPR Contributes to Adhesion, Invasion, and Biofilm Formation in Streptococcus agalactiae by Repressing Capsular Polysaccharide Production
Source: Microbiol Spectr. 2022 Jul 21;10(4):e02113-21. doi: 10.1128/spectrum.02113-21 (PMC9430516; doi:10.1128/spectrum.02113-21)
Supplement: Supplemental file 1 — Supplemental material. Download spectrum.02113-21-s0001.pdf, PDF file, 0.7 MB [file spectrum.02113-21-s0001.pdf]

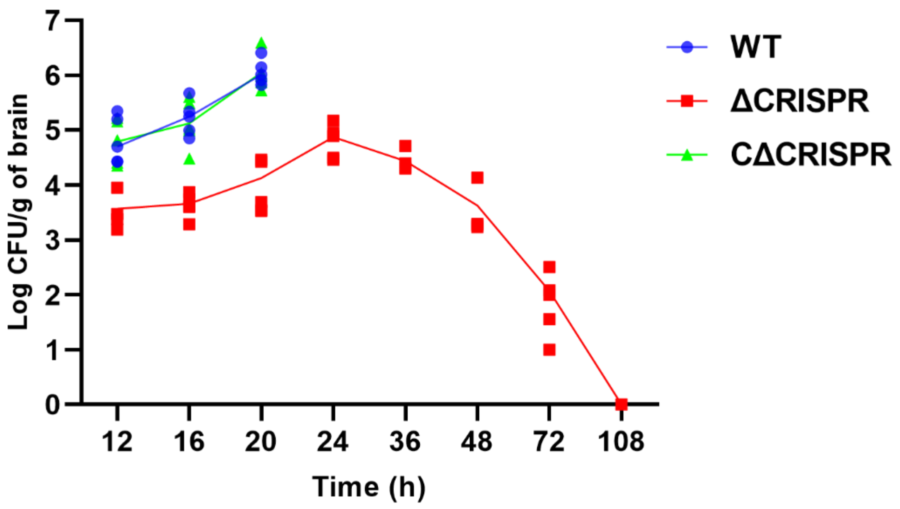

Fig. S1 The dynamic bacteria loads of the WT and ΔCRISPR strains in the brain tissue. The mice were sacrificed at 12 h, 16 h, 20 h, 24 h, 36 h, 48 h 72 h and 108 h postinfection, the brain samples were harvested, weighed and homogenized in PBS. The homogenates were serially diluted in PBS and plated to count the CFUs after overnight culture.

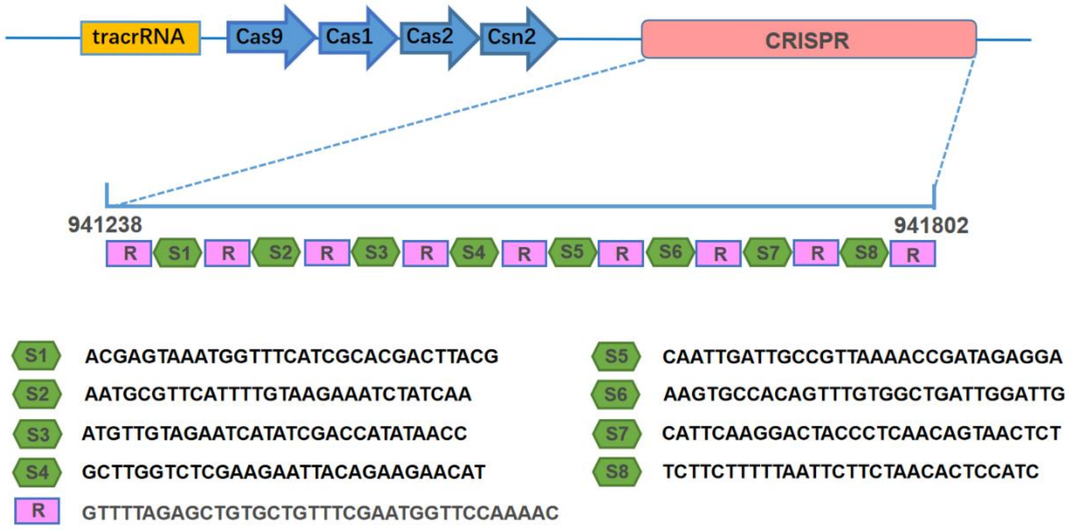

Fig. S2 Type II-A CRISPR-Cas system in *S. agalactiae* GD2008-001. Sequences are from 5′to 3′. The tracrRNA is in orange, the *cas* genes are in blue, the deleted targets (941238 bp-941802 bp) encoded in the CRISPR array are represented as pink rectangles, the repeats are purple rectangles, the spacers are green hexagons.

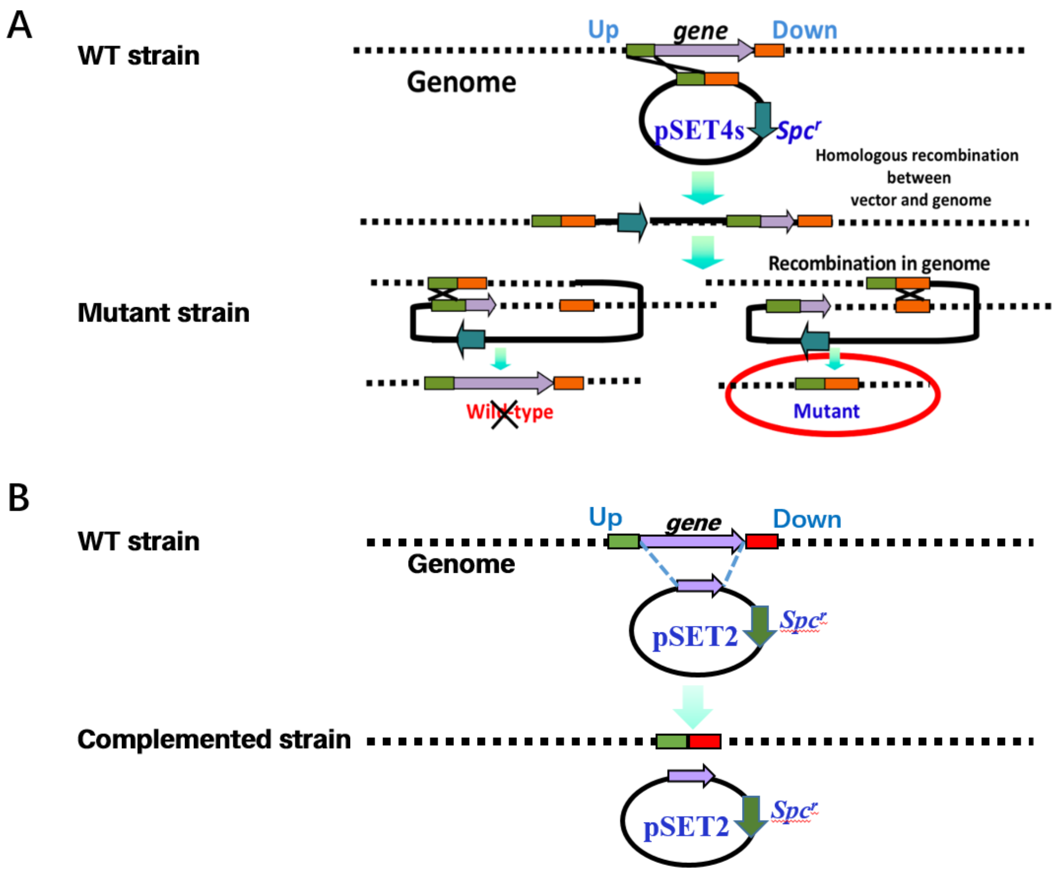

Fig. S3 The schematic diagram of the construction of the mutant (A) and complemented strain (B).

Table S1 The sequence identity of GD201008-001's CRISPR array compared to other GBS strains

NO.	Strain	Start position	End position	CRISPR length	Number of spacers	Percent of identity (%)
1	ZQ0910	942406	942970	564	8	100
2	HN016	942380	942944	564	8	100
3	TFJ0901	1538876	1539440	564	8	100
4	GX064	942126	942690	564	8	100
5	1173	957640	958599	959	14	94.40
6	WC1535	846922	847157	235	3	94.06
7	Sag37 CRISPR-1	507421	508249	828	12	0
	Sag37 CRISPR-2	1045243	1045938	695	10	87.62
8	FDAARGOS_512	183551	183652	101	1	86.54
9	GBS-M002	943680	943781	101	1	84.91
10	A909	986377	987336	959	14	81.76
11	GBS1-NY	968391	968559	168	2	81.40
12	GBS6	998542	998710	168	2	81.40
13	FDAARGOS_254	1582241	1582408	167	2	81.40
14	GBS2-NM	1003794	1003962	168	2	81.40
15	B509	1643626	1644916	1290	19	78.60
16	ILRI005 CRISPR-1	475530	475895	365	5	0
	ILRI005 CRISPR-2	933638	933871	233	3	78.48
17	SS1	941625	941924	299	4	77.71
18	SA111	971240	971605	365	5	77.66
19	COH1	871649	872015	366	5	77.45
20	BM110	963910	964737	827	12	77.44

21	NGBS572 CRISPR-1	486846	487674	828	12	0
	NGBS572 CRISPR-2	921980	922345	365	5	77.39
22	2603V_R	908164	909783	1619	24	77.08
23	NCTC13949	915056	916344	1288	19	76.92
24	B508	689200	690820	1620	24	76.87
25	FDAARGOS_670	811415	813034	1619	24	76.61
26	NCTC11930 CRISPR-1	1139022	1140047	1025	15	76.49
	NCTC11930 CRISPR-2	1540971	1541599	628	9	0
27	B111	201795	202886	1091	16	76.39
28	NCTC13947	1199171	1200327	1156	17	76.33
29	Sag27	1787785	1788875	1090	16	76.27
30	NGBS128	937050	937614	564	8	76.22
31	HU-GS5823	931914	933203	1289	19	76.20
32	NGB061	972927	974942	2015	30	76.19
33	GBS19	948459	949550	1091	16	76.16
34	SG-M25	1064343	1065169	826	12	76.12
35	BJ01	1376441	1378391	1950	29	76.08
36	Sag158	972049	973866	1817	27	76.08
37	SGEHI2015-113 CRISPR-1	471653	472281	628	9	0
	SGEHI2015-113 CRISPR-2	873875	874372	497	7	76.06
38	SG-M8	976520	977281	761	11	75.94
39	CJB111	942935	944091	1156	17	75.93
40	GBS11	988825	990378	1553	23	75.90
41	GBS28	985510	987525	2015	30	75.90
	FWL1402 CRISPR-1	525581	526143	562	8	0

42	FWL1402 CRISPR-2	927723	928616	893	13	75.89
43	GBS30	923009	924826	1817	27	75.81
44	SG-M6	900137	900964	827	12	75.77
45	NCTC8187	864910	865802	892	13	75.77
46	C001	936737	937893	1156	17	75.73
47	NJ1606	898798	899691	893	13	75.68
48	SS1168	888425	890110	1685	25	75.68
49	CNCTC 10_84 CRISPR-1	379940	380186	246	3	0
	CNCTC 10_84 CRISPR-2	913073	913900	827	12	75.59
50	874391	980031	980594	563	8	75.51
51	H002	921391	922482	1091	16	75.51
52	GBS7	939348	939977	629	9	75.51
53	09mas018883	940236	940931	695	10	75.51
54	S9968	1623683	1624576	893	13	75.43
55	SG-M4	828589	829548	959	14	75.42
56	FDAARGOS_669	1932932	1933693	761	11	75.34
57	515	879598	880425	827	12	75.34
58	32790-3A	908373	909068	695	10	75.30
59	GBS ST-1	965068	965555	487	6	75.21
60	GBS85147	856653	857281	628	9	75.21
61	B105	1083339	1083836	497	7	74.95
62	YZ1605	968280	968843	563	8	74.79
63	CU_GBS_98 CRISPR-1	464180	464808	628	9	0
	CU_GBS_98 CRISPR-2	866399	867028	629	9	74.79

64	SGEHI2015-107 CRISPR-1	471553	472181	628	9	0
	SGEHI2015-107 CRISPR-2	873772	874401	629	9	74.79
65	SG-M50 CRISPR-1	513652	514280	628	9	0
	SG-M50 CRISPR-2	915873	916502	629	9	74.79
66	SG-M163 CRISPR-1	513653	514281	628	9	0
	SG-M163 CRISPR-2	915874	916503	629	9	74.79
67	SG-M29 CRISPR-1	513652	514280	628	9	0
	SG-M29 CRISPR-2	915873	916502	629	9	74.79
68	SGEHI2015-95 CRISPR-1	513647	514275	628	9	0
	SGEHI2015-95 CRISPR-2	915859	916488	629	9	74.79
69	CU_GBS_08 CRISPR-1	503596	504158	562	8	0
	CU_GBS_08 CRISPR-2	905749	906444	695	10	74.79
70	SG-M1 CRISPR-1	513653	514281	628	9	0
	SG-M1 CRISPR-2	915875	916504	629	9	74.79
71	SG-M158 CRISPR-1	513653	514281	628	9	0
	SG-M158 CRISPR-2	915875	916504	629	9	74.79
72	PLGBS13	940451	941146	695	10	74.45
73	NEM316	951908	952801	893	13	74.29
74	CUGBS591 CRISPR-1	519847	520542	695	10	0
75	CUGBS591 CRISPR-2	1045941	1046504	563	8	74.28
76	SGEHI2015-25 CRISPR-1	509612	510240	628	9	0
77	SGEHI2015-25 CRISPR-2	911831	912394	563	9	74.14
78	B507 CRISPR-1	315631	316258	627	9	0
	B507 CRISPR-2	722837	723136	299	4	73.36
79	S73	509317	509945	628	9	0

80	SG-M50	513652	514280	628	9	0
81	138P	/	/	/	/	/
82	138spar	/	/	/	/	/
83	2012-845	/	/	/	/	/
84	GX026	/	/	/	/	/
85	ILRI112	/	/	/	/	/
86	NCTC8184	/	/	/	/	/
87	QMA0271	/	/	/	/	/
88	S13	/	/	/	/	/
89	S25	/	/	/	/	/
90	SA95	/	/	/	/	/
91	SA1	/	/	/	/	/
92	SA102	/	/	/	/	/
93	SA132	/	/	/	/	/
94	SA136	/	/	/	/	/
95	SA159	/	/	/	/	/
96	SA16	/	/	/	/	/
97	SA184	/	/	/	/	/
98	SA191	/	/	/	/	/
99	SA195	/	/	/	/	/
100	SA20	/	/	/	/	/
101	SA201	/	/	/	/	/
102	SA97	/	/	/	/	/
103	SA209	/	/	/	/	/
104	SA212	/	/	/	/	/

105	SA218	/	/	/	/	/
106	SA220	/	/	/	/	/
107	SA245	/	/	/	/	/
108	SA256	/	/	/	/	/
109	SA289	/	/	/	/	/
110	SA30	/	/	/	/	/
111	SA330	/	/	/	/	/
112	SA333	/	/	/	/	/
113	SA341	/	/	/	/	/
114	SA343	/	/	/	/	/
115	SA346	/	/	/	/	/
116	SA374	/	/	/	/	/
117	SA375	/	/	/	/	/
118	SA5	/	/	/	/	/
119	SA623	/	/	/	/	/
120	SA627	/	/	/	/	/
121	SA73	/	/	/	/	/
122	SA75	/	/	/	/	/
123	SA79	/	/	/	/	/
124	138P	/	/	/	/	/
125	SA85	/	/	/	/	/

Table S2 Differentially expressed genes of *cps* cluster between ΔCRISPR and TW detected by RNA-seq

Number	locus_tag	Gene and/or possible function	GenBank identification	Fold Change (ΔCRISPR/TW)
1	A964_1137	*cpsJ*, capsular polysaccharide biosynthesis protein CpsJ	AFS45902.1	2.58
2	A964_1145	*cpsC*, capsular polysaccharide biosynthesis protein CpsC	AFS45910.1	2.88
3	A964_1146	*cpsB*, capsular polysaccharide biosynthesis protein CpsB	AFS45911.1	2.50
